# EmbedDTI: Enhancing the Molecular Representations via Sequence Embedding and Graph Convolutional Network for the Prediction of Drug-Target Interaction

**DOI:** 10.3390/biom11121783

**Published:** 2021-11-29

**Authors:** Yuan Jin, Jiarui Lu, Runhan Shi, Yang Yang

**Affiliations:** 1 Center for Brain-Like Computing and Machine Intelligence, Department of Computer Science and Engineering, Shanghai Jiao Tong University, 800 Dong Chuan Rd., Shanghai 200240, China; aurora_yuan@sjtu.edu.cn (Y.J.); han.run.jiangming@sjtu.edu.cn (R.S.); 2 School of Chemistry and Chemical Engineering, Shanghai Jiao Tong University, 800 Dong Chuan Rd., Shanghai 200240, China; ari427@sjtu.edu.cn; 3 Key Laboratory of Shanghai Education Commission for Intelligent Interaction and Cognitive Engineering, Shanghai Jiao Tong University, 800 Dong Chuan Rd., Shanghai 200240, China

**Keywords:** drug-target interaction, graph convolutional network, molecular representation

## Abstract

The identification of drug-target interaction (DTI) plays a key role in drug discovery and development. Benefitting from large-scale drug databases and verified DTI relationships, a lot of machine-learning methods have been developed to predict DTIs. However, due to the difficulty in extracting useful information from molecules, the performance of these methods is limited by the representation of drugs and target proteins. This study proposes a new model called EmbedDTI to enhance the representation of both drugs and target proteins, and improve the performance of DTI prediction. For protein sequences, we leverage language modeling for pretraining the feature embeddings of amino acids and feed them to a convolutional neural network model for further representation learning. For drugs, we build two levels of graphs to represent compound structural information, namely the atom graph and substructure graph, and adopt graph convolutional network with an attention module to learn the embedding vectors for the graphs. We compare EmbedDTI with the existing DTI predictors on two benchmark datasets. The experimental results show that EmbedDTI outperforms the state-of-the-art models, and the attention module can identify the components crucial for DTIs in compounds.

## 1. Introduction

The detection of drug-target interactions (DTIs) is a key step in drug development and drug repositioning. In recent decades, high-throughput screening (HTS) experiments have greatly accelerated the identification of DTIs. However, HTS experiments are costly and laborious, which cannot meet the need for revealing DTIs for millions of existing compounds and thousands of targets [1,2]. Therefore, there is a strong motivation to establish computational tools for predict DTIs automatically [3].

The rapid increase of DTI data in public databases, such as ChEMBL [4], DrugBank [5], and SuperTarget [6], has enabled large-scale in silico identification of DTIs. The computational methods mainly fall into three categories, namely docking-based, similarity search-based and feature-based.

For docking-based methods, the three-dimensional structures of target proteins are used to simulate the binding position and orientation by considering various transitions and rotation of the ligands to gain different binding conformations [7,8,9,10]. These methods minimize the binding free energy by designing a scoring function to predict effective protein-ligand binding. The efficacy of docking methods depends on protein 3D structure information, while 3D structures of many target proteins are still unknown, such as GPCRs [11]. Moreover, the simulation of the docking process is relatively time-consuming, and can only be used when the prediction scale is small.

The similarity search-based methods assume that small molecular compounds with similar structures or physic-chemical properties can act on targets with the same or similar properties [12,13,14,15]. Thanks to the rapid increase of drug information and target annotation in public databases, similarity search-based methods have been widely used in recent years. However, they only work for predicting the binding to proteins similar to known targets and fail to recognize DTIs for novel targets.

In contrast to docking-based and similarity search-based methods, feature-based methods use various types of features extracted from drug compounds and target proteins and mainly adopt machine-learning models to predict DTI relationships. Feature-based methods can be roughly divided into two types. The first type adopts collaborative matrix factorization techniques [16,17,18]. This type of method decomposes the known drug-target relationship matrix into two low-dimensional feature matrices representing drug and target protein, respectively. Based on the drug and target feature matrices, similarity matrices of drugs and targets can be estimated by taking the inner product of the feature vectors. Given the drug-target relationship matrix as well as the two similarity matrices, potential DTIs can be inferred. For instance, DTINet predicts novel drug-target interactions from heterogeneous networks [19], by integrating diverse drug-related information. DTINet focuses on learning a low-dimensional vector representation of features, which accurately explains the topological properties of individual nodes in the heterogeneous network, and then makes a prediction based on these representations via a vector space projection scheme.

The second type of feature-based methods use extracted feature descriptors of drug compounds and target proteins respectively, and models the DTI prediction as a binary classification (interaction exists or not) or regression problem (the output is binding affinity) [20,21,22]. Molecular fingerprints are commonly used as descriptors of drug substructures, while composition, transition, and distribution (CTD) are commonly used as protein descriptors.

Feature-based methods have been more widely used in recent years, as they have few limitations on the input information source. However, their performance relies heavily on feature representation. In the existing drug and target descriptors, molecular structural information is often absent, thus leading to unsatisfactory prediction results.

As deep neural networks (DNNs) have achieved great success in automatic feature learning for image and sequence data, some deep learning models have also been proposed to predict the binding affinities between drugs and targets. By inputting raw drug and target protein data, DNNs can extract useful information for prediction. For example, DeepDTA employs a convolutional neural network (CNN) to extract local sequence patterns as a high-level feature representation for drug-target binding affinity prediction [23]. Another method called DeepConv-DTI [24] also adopts CNNs. In contrast to DeepDTA, which mainly focuses on protein kinases, DeepConv-DTI was trained on a larger scale dataset with diverse types of proteins. Later, a DTI model named GraphDTA [25] was proposed to predict drug-target binding affinities, which is a state-of-the-art method for kinase-type target proteins. Compared with DeepDTA [23], WideDTA [26], PADME [27], and MT-DTI [28], which represent drug compounds as strings to extract feature vectors, GraphDTA represents drugs in the form of graph and use graph convolutional network (GCN) for feature learning.

Despite the recent progress, there is still large room to improve the feature representation of drugs and target proteins to enhance DTI prediction. In this study, we propose a new method, EmbedDTI, which leverages embedding vectors for protein sequences and graph representations for both atoms and substructures of compounds to enhance the molecular representations. We evaluate the performance of our model on two benchmark datasets, the Kinase dataset Davis [29] and KIBA dataset [30], and compare results with a series of the existing models, including KronRLS [14], SimBoost algorithms [15], DeepDTA [23], WideDTA [26], and GraphDTA [25]. EmbedDTI obtains the lowest mean square error (MSE) and the highest concordance index (CI). Furthermore, we perform a case study of inhibitor design for K-Ras target. The candidate compounds with high binding capability identified by EmbedDTI show stable docking with K-Ras target.

## 2. Materials and Methods

### 2.1. Metrics of Binding Affinity

Binding affinity provides specific information about the interaction between drug-target (DT) pairs. It can be measured by metrics such as the half-maximal inhibitory concentration (IC${}_{50}$), dissociation constant (K${}_{d}$), inhibition constant (K${}_{i}$), and association constant (K${}_{a}$). IC${}_{50}$ represents the concentration of the drug or inhibitor required to inhibit half of the specified biological process (or a component in the process such as enzymes, receptors, cells, etc.). K${}_{i}$ reflects the inhibitor’s inhibitory strength to the target. The smaller the value, the stronger the inhibitory ability. K${}_{d}$ reflects the affinity of the drug compound to the target. The smaller the value, the stronger the affinity. In some cases, it is equivalent to K${}_{i}$. K${}_{a}$ is the reciprocal of K${}_{d}$. Thus, the larger the value of K${}_{a}$, the stronger the binding affinity. Following the practice of previous studies [15], we adopt the log-transformed K${}_{d}$ (Equation (1)) as the model output.

(1) $$pK_{d}=-\log_{10}\left(\frac{K_{d}}{1e9}\right)$$

### 2.2. Datasets

In this paper, we evaluate our model on two benchmark sets, the Kinase dataset Davis [29] and KIBA dataset [30], which were used in DeepDTA [23], WideDTA [26], PADME [27], MT-DTI [28], and GraphDTA [25]. Table 1 shows the overview of these two datasets.

The Davis dataset collects clinically related kinase protein families and related inhibitors with their respective dissociation constant (K${}_{d}$) values, while KIBA is a more general dataset and much larger than Davis. In Davis, only K${}_{d}$ is used to measure the biological activity of kinase inhibitors; while KIBA combines K${}_{i}$, K${}_{d}$, and IC${}_{50}$ to obtain KIBA scores of protein families and related inhibitors. The EmbedDTI model performance is assessed on these two datasets, respectively.

### 2.3. Corpus for Pretraining Protein Embeddings

Instead of using traditional one-hot encoding for target proteins, EmbedDTI generates a pre-trained amino acid embedding matrix to represent target proteins. Here we use the UniRef50 database [31] as the corpus for pretraining, including 48,524,161 amino acid sequences.

## 3. Methods

### 3.1. Model Overview

**Figure 1:**
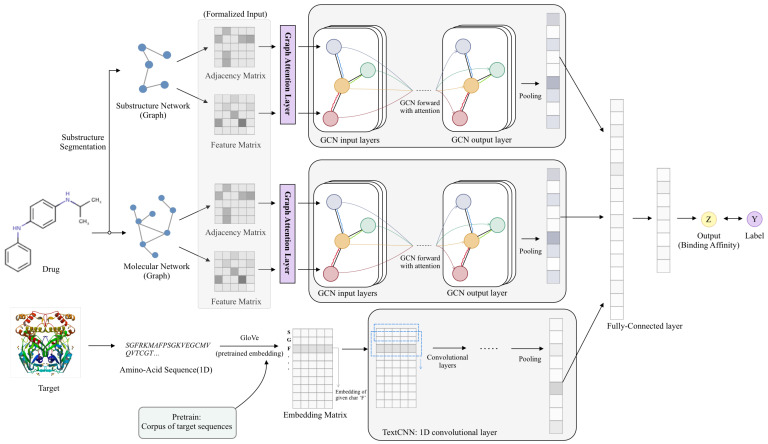
Model architecture. For protein sequences, we leverage GloVe for pretraining the feature embeddings of amino acids and feed them to a CNN model for representation learning. For drugs, we construct two levels of graphs to represent compound structural information, namely the atom graph and substructure graph. Graphs of different levels provide an embedding representation vector respectively through attention and several GCNs. Three embedding vectors are concatenated to output the binding affinity of the drug-target pairs through several fully connected layers.

Figure 1 shows the architecture of EmbedDTI. It consists of three major components, namely initial feature extraction, feature learning, and classification.

The raw inputs of EmbedDTI are amino acid sequences of target proteins and SMILES of drug compounds. In the initial feature extraction part, the GloVe algorithm [32] is employed to obtain the pre-trained embedding representations of amino acids. For drugs, we convert their SMILES sequences into two graph structures to retain as much structural information as possible for feature learning. One graph consists of atoms as nodes and bonds between atoms as edges, which represents information about individual atoms and their neighbors. The other one is a graph of substructures, i.e., each node denotes a substructure in the compound instead of an atom. According to the graph structures, we obtain the adjacency matrix. For each node of the graph, some chemical and data structural features are extracted to form a feature matrix.

In the feature learning part, for target proteins, we input their pre-trained embedding vectors into CNN blocks to obtain high-level abstract sequence representations. For each drug, we obtain two feature embeddings from the two kinds of graphs. Each graph corresponds to an adjacency matrix and the nodes’ feature matrix, which are fed into a GCN network for training. A max-pooling layer is used to aggregate the features of every node to obtain an embedding representation of the whole graph. In addition, we add a scaled dot-product attention layer before the GCN network for atom and substructure branch to help learn the relative importance of each node (atom or substructure).

After feature learning, we concatenate the three feature vectors into a whole vector and feed it into several fully connected layers to obtain the binding affinity scores of drug-target pairs.

Details of the three components are described in the following sections.

### 3.2. Initial Feature Extraction

#### 3.2.1. Input Features of Target Proteins

In EmbedDTI, the input features for proteins are extracted from amino acid sequences. To obtain good representation for amino acid sequences, we leverage word embedding techniques in natural language processing to perform a pretraining on a large protein database, UniRef50, and obtain embedding vectors for amino acids. The GloVe [32] model is used to obtain embeddings for amino acids. GloVe is an unsupervised model that can learn a fixed-length feature vector representation from the variable-length text, which is based on the aggregated global word-word co-occurrence statistics of the corpus. Here we consider each amino acid as a word.

#### 3.2.2. Input Features of Drugs

Chemical compounds are usually represented as graph-structured data in computers, where the vertexes and edges correspond to atoms and chemical bonds, respectively. An atom-based graph can represent structural information between atoms in short distances but ignores functional groups in compounds, which play important roles in determining the properties and reactions of compounds. For example, a single atom in a benzene ring can learn information about its neighboring atoms, but it is difficult to learn about the structure of the entire ring as a whole. Therefore, we define substructures and convert the original chemical graph into a higher-level graph of substructures, in which the nodes and edges correspond to substructures and connections between substructures, respectively.

EmbedDTI extracts information from both the atom graph and substructure graph and then combines them for the final prediction. The two levels of graphs are described below.

#### Atom-Level Representation

Atom graphs can be converted from SMILES strings, a common description of chemical compounds (SMILES: simplified molecular input line entry specification, a specification that uses ASCII strings to describe the molecular structure [33]), which are publicly available. To extract atom information, we use the open-source chemical information software RDKit [34]. Each node is represented as a one-hot feature vector containing eight kinds of information, i.e., the atomic symbol, the degree of the atom in the molecule, the total number of Hs (explicit and implicit) the atom is connected to, the number of implicit Hs the atom is connected to, the total valence (explicit + implicit) of the atom, the charge of the atom, whether or not the atom is aromatic, and whether or not the atom is in a ring. Finally, we obtain a 101-dimensional one-hot vector for each atom.

#### Substructure-Level Representation

**Figure 2:**
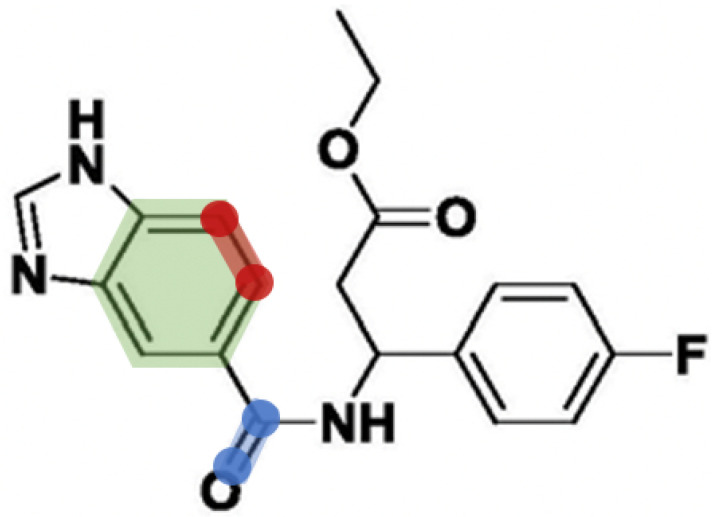
Two different types of bonds. The red marked one is a bond in a ring, while the blue marked one is a bond outside any ring.

A major limitation of the atom graph is that it treats all edges equally and extracts information from individual vertexes, while atoms and related edges often function in groups. Take Figure 2 as an example. The bond between blue nodes is important for the entire molecule, while the bond between red nodes is meaningless if segmented out separately from the ring structure.

**Figure 3:**
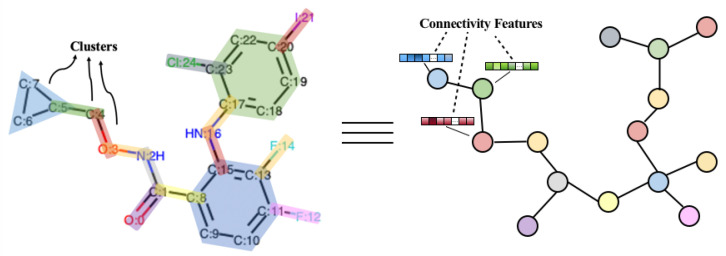
An example of substructure segmentation. The left graph is the atom-level graph, where substructures are marked by different colors. The right one is the substructure-level graph, where each substructure is denoted by a single node in the graph.

Here we propose a segmentation method and obtain a complete set of substructures to ensure that all compounds in the database can be composed of substructures in the set. As illustrated in Figure 3, we segment the whole graph into a tree of substructures. A substructure is either a cyclic substructure that has less than 3 atoms shared with other rings, or a pair of atoms linked by a bond that does not belong to a ring [35]. In this way, molecular compounds can be regarded as topological graphs connected by substructures. The substructure segmentation algorithm is formulated in Algorithm 1. The molecule objects are obtained by the Chem.MolFromSmiles function in RDKit. $V_{1}$ and $V_{2}$ involve independent bonds and simple rings, respectively. Bonds are extracted from the GetBonds function while simple rings are extracted from the Chem.GetSymmSSSR function. Finally, we have a vocabulary of bonds that are not in any ring and independent rings with less than 3 atoms shared with other rings.
**Algorithm 1** Segmentation of substructures for molecule G=(V,E)**Input:** SMILES strings of compounds**Output:** Vocabulary of substructures *C*Get molecule object from SMILESNumber the atoms in the compound moleculeInitialize: vocabulary of substructures C=⌀Construct $V_{1}$← the set of bonds ∈EConstruct $V_{2}$← the set of simple rings of *G***for** each bond $e_{i}$ in $V_{1}$ **do**    **if** $e_{i}$ does not belong to any ring **then**        add $e_{i}$ to the vocabulary of substructures *C*    **end if****end for****for** each ring $r_{i}$ in $V_{2}$ **do**    **for** each ring $r_{j}$ in $V_{2}$ **do**        $inter=r_{i}\cap r_{j}$        **if** the length of inter≥ 3 **then**           tmp← merge $r_{1},r_{2}$ to one unique ring           $r_{i}$←tmp           $r_{j}$←tmp        **end if**    **end for****end for**remove duplicate substructures from $V_{2}$add each substructure in $V_{2}$ to the vocabulary of substructures *C***return** vocabulary of substructures *C*

Similar to the atom-level graph, node information is also extracted in a substructure-level graph. Here, we extract five kinds of structural information based on graph theory for each substructure, (i) the number of atoms, (ii) the number of edges connected to the substructure, (iii) the number of hydrogen atoms (explicit and implicit), (iv) whether or not it contains a ring, (v) whether or not it contains a non-ring bond. And then, each substructure is represented as a 35-dimensional one-hot vector which is the initial feature representation.

### 3.3. Feature Learning Using Deep Neural Networks

#### 3.3.1. Target Feature Learning via CNN

As mentioned, we use GloVe to obtain pre-trained embeddings for each amino acid $e_{i}$ (0≤i≤L, where *L* represents the maximum length of the protein sequence), then we feed the embedding matrix *E* into a deep convolutional neural network (CNN) for further feature learning. We employ a three-layer 1D CNN. The CNN model extracts local sequence features via convolution kernels operated in the neighborhood of residues. The CNN is followed by two fully connected layers to yield a 128-dimensional representation vector *P* for each protein sequence.

#### 3.3.2. Drug Feature Learning via GCN

CNNs have not only achieved great success in computer vision and natural language processing but also showed good performance in various graph-related learning tasks, where the nodes are in non-Euclidean spaces. In particular, graph convolution networks (GCNs) [36] aim to capture local correlations of signals on graphs. As drugs can be represented in the form of graphs, GCNs are employed to learn features from drugs in EmbedDTI.

Formally, for a graph G=(V,E), where *V* is a set of nodes and *E* is a set of edges. Each node *i* has its characteristics $x_{i}$, which can be represented by a matrix $X\in R^{N\times d}$, where *N* represents the number of nodes and *d* represents the number of features of each node, i.e., the dimensionality of the feature vectors. The connecting relationship between nodes forms an N×N-dimensional adjacency matrix *A*. $X\in R^{N\times d}$ and $A\in R^{N\times N}$ are the input of one GCN layer. The propagation between layers of GCN can be formulated in Equation (2).

(2) $$H^{\left(l+1\right)}=\sigma \left({\tilde{D}}^{-\frac{1}{2}}\tilde{A}{\tilde{D}}^{-\frac{1}{2}}H^{\left(l\right)}W^{\left(l\right)}\right),$$

where $\tilde{A}$ is the adjacency matrix plus self-connected edges, $\tilde{D}$ is the degree matrix of $\tilde{A}$, $H^{\left(l\right)}$ represents the characteristics of the l-th layer. σ is an activation function, such as ReLU. For the input layer, $H^{\left(0\right)}$ is equal to *X*.

(3) $$H^{\left(0\right)}=W\times X,$$

where *W* is an attention weight matrix.

**Figure 4:**
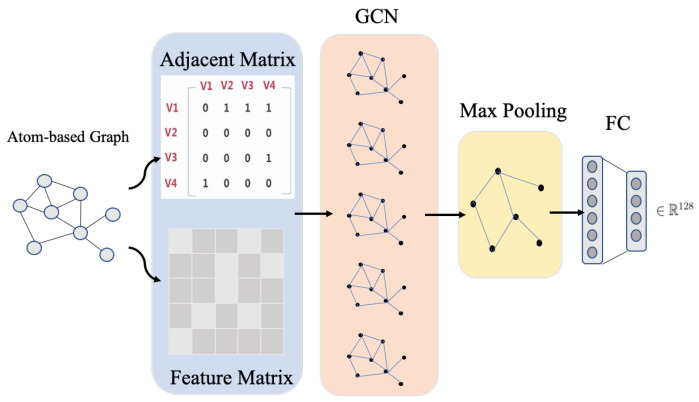
The graph feature learning via GCN. Taking the adjacency matrix and feature matrix of a graph as the input, the node-level representation is obtained after convolution operation. Then, the node-level representation is passed through a max-pooling layer to obtain the graph-level representation. Finally, the graph-level representation matrix is expanded, and a 128-dimensional vector is obtained through several fully connected layers.

GCN model learns the node-level outputs $Z\in R^{N\times F}$, where *F* is the number of filters. To obtain the graph-level representation, we add a max-pooling layer after GCN layers. Similar to the pooling operation in traditional CNN, max-pooling is a reasonable downsizing to a graph. Figure 4 shows the GCN learning process for an atom graph.

**Figure 5:**
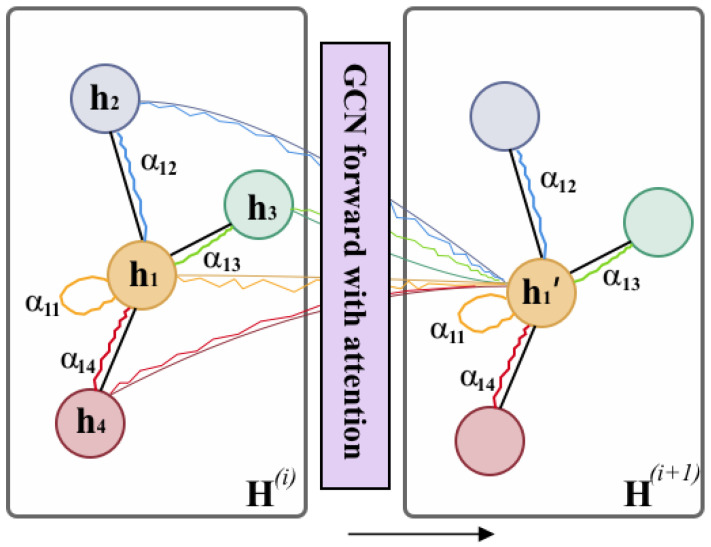
GCN forward layer with attention. The attention module will consider each pair of nodes and assign them with attention weight $\alpha_{ij}$, which indicates the node *j* has $\alpha_{ij}$-weighted influence on node *i* during the propagation.

In addition, in the propagation step of GCN, we add a node-wise attention layer to help learn the relative importance of each node (atom or substructure). At this time, $H^{\left(0\right)}$ is shown in Equation (3). Figure 5 illustrates this process.

### 3.4. Prediction Model

After feature learning, we have obtained three 128-dimensional feature vectors *P*, $A_{m}$ and $C_{q}$, which are the representations for target proteins, atom-level drug molecules, and substructure-level drug molecules, respectively. We concatenate them as a vector *T* (Equation (4)) and pass them into three fully connected layers to obtain the binding affinity scores of drug-target pairs.

(4) $$T=P⊕A_{m}⊕C_{q}\in R^{384}$$

## 4. Results

### 4.1. Experimental Settings

We assess the performance of EmbedDTI on two benchmark sets, the Kinase dataset Davis [29] and KIBA dataset [30]. For a fair comparison, we use the same data division strategy as DeepDTA [23], which randomly divided the datasets into 6 equal parts. One for independent test and others for training, where 5-fold cross-validation within the training set is performed to search optimal hyper-parameters. For each hyper-parameter, we use a grid search to narrow the search range to the neighborhood of the optimal parameter and then perform a refined search.

In the feature learning part, for proteins, we use three convolutional layers with different filter sizes. And the GCNs for learning atom-based graphs and substructure-based graphs of compounds also contain three graph convolutional layers. The parameter settings are shown in Table 2.

### 4.2. Evaluation Metrics

Since we consider DTI as a regression problem to predict binding affinity between drug-target pairs, we use mean squared error (MSE) as the loss function. MSE measures the difference between the predicted value (*P*) and the true value of the target variable (*Y*). The smaller the MSE, the closer the predicted value to the true value, and vice versa. Let *N* denote the number of samples, the MSE is defined in Equation (5).

(5) $$\mathrm{MSE}=\frac{1}{N}\sum_{i=1}^{N}{\left(y_{i}-p_{i}\right)}^{2}$$

Another metric we use to evaluate the performance is the concordance index (CI), which was proposed by [14]. CI is used to calculate the discrimination between the predicted value and the true value of the model, as defined in Equation (6),

(6) $$CI=\frac{1}{Z}\sum_{\delta_{x}>\delta_{y}}h\left(b_{x}-b_{y}\right),$$

where $b_{x}$ is the predicted binding affinity relative to the real larger binding affinity $\delta_{x}$, $b_{y}$ is the predicted binding affinity relative to the real smaller binding affinity $\delta_{y}$, h(x) is a step function shown in Equation (7), and *Z* is a normalization constant used to map the value to the interval [0, 1]. The CI indicator measures whether the predicted affinity values of two randomly selected drug-target pairs maintain a similar relative order in the real dataset. The larger the CI value, the better the result.

(7) $$h\left(x\right)=\left\{\begin{array}{ccc} 0 & \; & \mathrm{if}\;x<0\\ 0.5 & \; & \mathrm{if}\;x=0\\ 1 & \; & \mathrm{if}\;x>0 \end{array}\right.$$

In addition, we compute two correlation coefficients, Pearson and Spearman, for correlation analysis, as formulated in Equations (8) and (9).

(8) $$\rho_{X,Y}=\frac{cov(X,Y)}{\sigma_{X}\sigma_{Y}},$$

where *X* and *Y* represent the true value and predicted value, respectively. cov(X,Y) represents the covariance matrix of *X* and *Y*. $\sigma_{X}$ and $\sigma_{Y}$ are the standard deviations of *X* and *Y*, respectively.

(9) $$\rho_{spearman}=1-\frac{6\sum_{i=1}^{n}{\left(x_{i}-y_{i}\right)}^{2}}{n(n^{2}-1)},$$

where $x_{i}$ and $y_{i}$ denote the ranks of *X* and *Y* in the true values and predicted values for the *i*-th sample, respective, and *n* is the number of elements.

### 4.3. Results on Davis Dataset

To assess the performance of EmbedDTI, we compare it with five state-of-the-art models as listed below.

- KronRLS [14]. It adopts Smith-Waterman algorithm to compute similarity between proteins and the PubChem structure clustering server to compute similarity between drug compounds. Then it uses a kernel-based method to calculate Kronecker products and integrates multiple heterogeneous information sources within a least squares regression (RLS) framework.
- SimBoost algorithms [15]. Its representation of proteins and drug compounds is the same as that of KronRLS. It constructs features for drugs, targets, and drug-target pairs, and extracts the feature vectors of drug-target pairs through feature engineering to train a gradient boosting machine to predict binding affinity.
- DeepDTA [23]. It encodes the original one-dimensional protein sequences and SMILES sequences. The encoded vector is passed through two independent CNN blocks to obtain the corresponding representation vector, and after concatenating, the predicted binding affinity is output through the fully connected layer.
- WideDTA [26]. It adds protein domains and motifs, and maximum common substructure words based on DeepDTA, a total of four parts of the original information training model.
- GraphDTA [25]. It uses TextCNN to perform feature learning on one-dimensional protein sequences. For the SMILES sequence, it uses four models of GCN, GAT, GIN, and GAT_GCN to obtain the representation vector of SMILES sequence.

In addition, we perform an ablation study on EmbedDTI by comparing three variants of EmbedDTI, i.e., EmbedDTI_noPre, EmbedDTI_noSub, and EmbedDTI_noAttn.

- EmbedDTI_noPre: no pretraining for protein sequences.
- EmbedDTI_noSub: no substructure graph representation for drug compounds.
- EmbedDTI_noAttn: no attention module in the GCN.

Table 3 shows the MSE and CI scores on the independent Davis test dataset compared with 5 baseline models. As can be seen, EmbedDTI achieves the lowest MSE and the highest CI, which decreases MSE by 9.5% and increases CI by 2.3% compared with the start-of-the-art method GraphDTA. The performance gain can be attributed to the following three factors.

First, we use graphs to represent compounds, which retain more structural information compared with the methods based on raw sequences. Moreover, we represent compounds by two kinds of graphs, involving both structural and functional information on the atom and substructure levels, rather than only one graph that is used in most existing methods like GraphDTA.

Second, the attention mechanism in GCN helps learn important information of nodes (atom or substructure). By outputting the attention score for each node, we can observe the focus of the model for predicting DTI.

**Figure 6:**
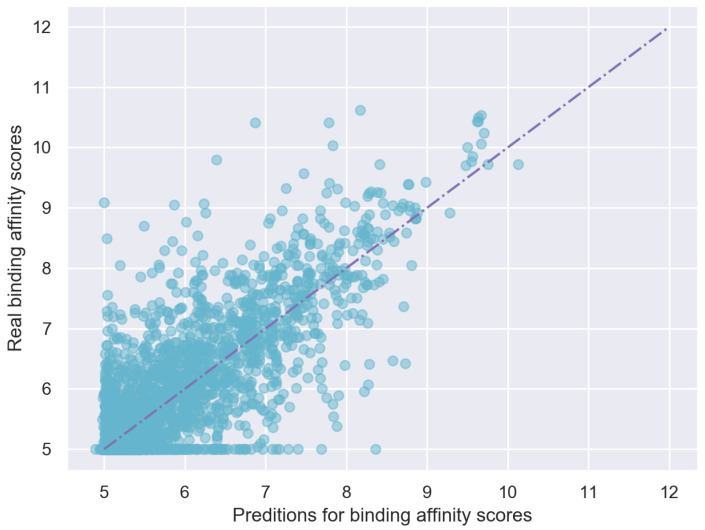
Predicting scores VS. Real scores on Davis test dataset.

Third, pretraining is used to improve the representation of target sequences by introducing some prior background knowledge, which also improves the overall performance of EmbedDTI. The predicted binding affinities and true binding affinities are plotted in Figure 6. It can be observed that most points are close to the line x=y.

### 4.4. Results on KIBA Dataset

For the KIBA dataset, we compare the performance of EmbedDTI with the same baseline models described in the previous section. Table 4 shows their MSE and CI scores. As can been seen, the performance of these models has the same trend as on Davis dataset, although KIBA is much larger than Davis. The graph-based representation of drugs improves the performance greatly (0.268 vs. 0.058 comparing WideDTA and GraphDTA on MSE). The benefit of two-level graphs is not as obvious as on Davis, while CI is increased by 0.013 in EmbedDTI compared with GraphDTA.

**Figure 7:**
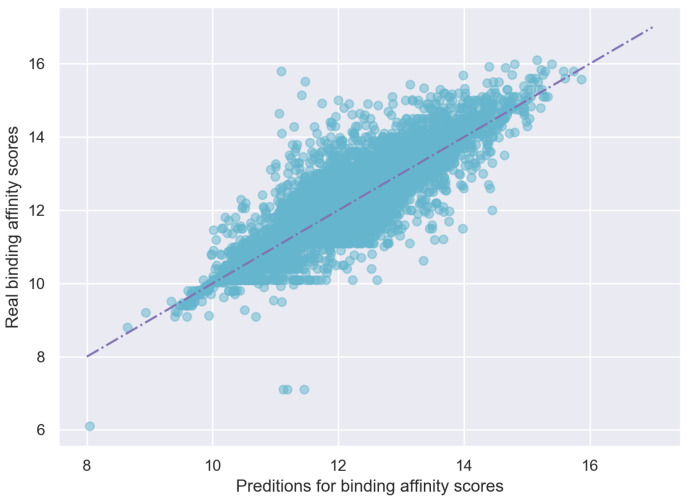
Predicting scores vs. Real scores on KIBA test dataset.

The predicted scores and true scores are plotted in Figure 7, which shows that the predicted values of EmbedDTI are close to the real values.

In summary, on both the two datasets, EmbedDTI achieves the lowest MSE value and the highest CI value. In particular, the comparison with baseline models suggest that both protein and drug representations contribute to the performance enhancement.

## 5. Case Study: Inhibitor Design for K-Ras Target

### 5.1. Molecular Evaluation Metrics

We use the following metrics of molecules to evaluate the results.

(i) Quantitative estimate of drug-likeness (QED) [37]. Quantitative estimate of drug-likeness (QED) is a widely used metric in drug discovery based on eight important properties that were previously used to assess drug-likeness of candidate molecules, including molecular weight (MW), octanol–water partition coefficient (ALOGP), number of H-bond donors (HBD), number of H-bond acceptors (HBA), molecular polar surface area (PSA), number of rotatable bonds (ROTB), number of aromatic rings (AROM), and number of structural alerts (ALERTS). The QED score (scaled between 0 and 1) was designed for molecular evaluation that to what extent a candidate is close to the corresponding average values in the QED benchmark set of 771 approved oral drugs. A higher QED score indicates more similar to the properties of drugs.

(ii) Synthetic accessibility (SA) [38] is the assessment from the structural perspective, which quantifies the synthetic accessibility of drug-like molecules by examining the fragment or submolecular features. Penalty will be added if the given molecules contain complex ring and too many atoms compared with normal drugs.

(iii) Docking score. In our experiment, the complex of interest is formed by compound ligand and protein receptor. The docking prediction is thus the binding affinity between ligand and receptor. Current docking can achieve good accuracy of affinity prediction and can replace unnecessary biomedical assays to reduce overheads. Here, the docking is used as an assessment tool for candidates after virtual screening based on the belief that docking can provide accurate computational approximation of ground truth binding affinity. This serves as the bioactivity evaluation of candidate molecules.

### 5.2. Implementation Details and Results

In this section, a case study is performed to design inhibitors of K-Ras target by molecule generation and virtual screening. K-Ras protein is made by KRAS gene and relays the signals from outside the cell to inside the cell, i.e., the nucleus. The K-Ras protein can be classified into the GTPase family. Small mutation of K-Ras may lead to serious illness such as colorectal cancer and lung cancer, as K-Ras is related to oncogene and somatic KRAS mutations are found at high rates in these cancers. K-Ras is an important drug target, while the lack of binding site information has hindered the pharmaceutical development. This case study implements a computational pipeline of drug design for K-Ras target using the proposed EmbedDTI that has been trained on the KIBA dataset in Section 4.4, because KIBA dataset is much larger than the Davis dataset.

First, we employ generative model MARS to obtain a set of molecules ${\left\{x_{i}\right\}}_{i=1}^{N}$ (N=5000) to be screened. The number of heavy atoms (non-hydrogen atoms) of these molecules are controlled to be within 40. In this work, we use a molecular generative model called MARS (MArkov MoleculaR Sampling) [39] to sample candidate molecules for further virtual screening. MARS employs Markov chain Monte Carlo (MCMC) sampling to perform several edition over chemical structure. We choose the generative model due to its good evaluation performance to generate diverse and novel species with good coverage of chemical space. In terms of run time configuration, we choose to generate a set of molecules ${\left\{x_{i}\right\}}_{i=1}^{N}$ (N=5000) after 1000 steps of edition. Other settings are used by default. Large molecular structure can lead to extra bias, and it is unrealistic for commonly used drugs. These molecules are in the format of SMILES sequence representation. For the set of molecules, we feed them and the sequence of K-Ras protein into the model EmbedDTI to make prediction of possible interaction. Then each molecule will be attached with a prediction score $P_{i}$, indicating the binding affinity with K-Ras receptor by EmbedDTI. Then we perform virtual screening to the molecular set. Specifically, the molecular population is ranked by their prediction score $P_{i}$ and only the top 10 molecules with highest affinity score (predicted ${\mathrm{pK}}_{d}$, the higher the better) and docking score (computed by SMINA) below a threshold are selected for further analysis. After that, we use the RDKit software to embed these molecules into three-dimensional space using conformer embedding methods.

For each generated molecule, we filter out its molecular conformers with very high energy (beyond the energy scale of normal molecules) as they are bad embedding results to exist in real case. Most of these bad cases are due to impossible bond angle, too short distance of non-bond pair of atoms, or illness of dihedral. Then the embedding process is repeated until acceptable structure appears. Otherwise, we discard this molecule.

**Figure 8:**
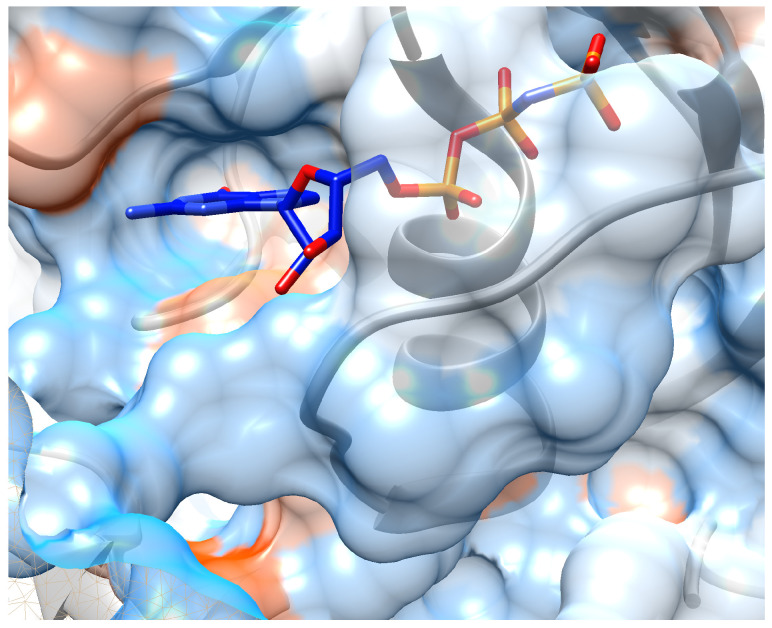
Crystal structure of ligand: phosphoaminophosphonic acid-guanylate ester binding into chain A of K-Ras. Protein sequences are colored as grey ribbon and its hydrophobic surface are also shown around the ribbon.

Finally, the embedded molecular structures are saved in SDF format files and fed into the SMINA docking simulation. We download the crystal structure of K-Ras target from Protein Data Bank (PDB) with PDB ID: 6FA2. And the binding pocket is kept the same as the ligand in complex of chain A: the center of search box is at (64, 108, 0) with size of each direction as (25, 30, 22). The unit is angstrom ($10^{-10}$ m). During docking, the ‘EXHAUSTIVENESS’ of search is set to be 16 and hydrogen atoms are added for docking. The best docking mode (with lowest affinity energy) is output as candidate binding pose for given molecule. We obtain a docked structure for each candidate molecule in the specified binding pocket of K-Ras receptor chain A, along with the binding affinity energy (in kcal/mol) output by SMINA (shown in Figure 8). The visualization is based on these coordinate files of receptor and candidate compounds using Chimera. SMILES and candidate molecules are shown in Table 5 as well as their corresponding scores in Table 6.

We use three performance metrics, i.e., quantitative estimate of drug-likeness (QED), synthetic accessibility (SA), and docking score. The QED score (ranging from 0 to 1) was designed for molecular evaluation that to what extent a candidate is close to the corresponding average values in the QED benchmark set of 771 approved oral drugs. A higher QED score indicates a larger similarity to the property of these drugs and thus more drug-like. The synthetic accessibility (SA) is an assessment metric from the structural perspective by quantifying the synthetic accessibility of drug-like molecules by examining the fragment or submolecular features. Penalty will be added if given molecules contain complex ring and too many atoms way more than normal drugs. Docking score approximates the binding affinity between ligand compounds and receptors, which serves as a bioactivity evaluation for candidate molecules.

As shown in Table 6, all the drug-target complexes have acceptable free energy decrease. The QED scores seem not very high, perhaps because the QED benchmark set is a relatively small set, covering only 771 approved oral drugs, while both the SA score and docking score look good. The high SA scores suggest that the candidate molecules are very similar to real molecules, and the low docking scores show a stable binding state.

In addition, we visualize the binding pose of candidate molecules into K-Ras receptor. We use UCSF Chimera as the visualization tool and display possible interaction between ligand and receptor. Chimera is open-sourced and functional for chemical or biological analysis. Visualizations of ten candidate molecules are shown in Supplementary Materials Figures S1–S10. From these figures, good shape complementarity can be observed between the candidate drug compounds and the K-Ras protein.

These results show that the candidate molecules are approachable and promising to put into assay validation in synthesis sense, and also suggest that EmbedDTI can be a useful tool for drug screening.

## 6. Investigation on the Model Attention

**Figure 9:**
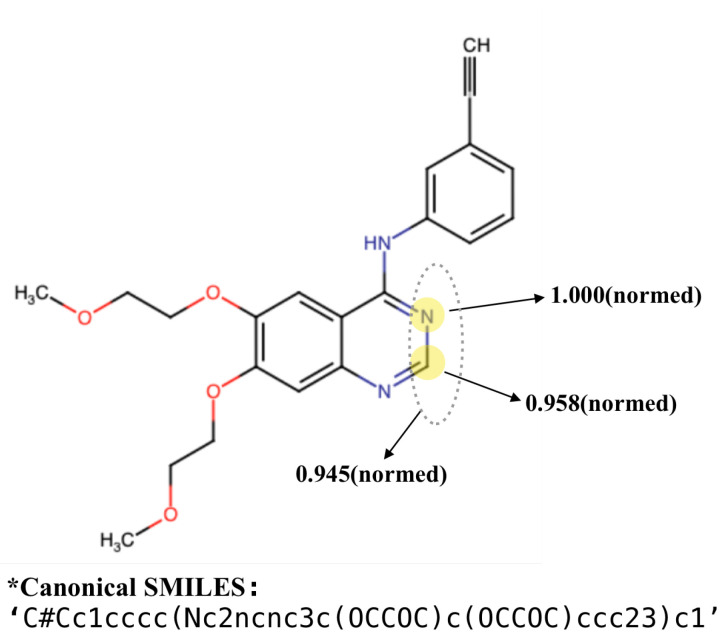
A fused nitrogen heterocyclic compound molecule with 29 atoms and 17 substructures (processed by partition algorithm). By attention output, the two atoms, C(id = 13) and N(id = 14) with highest normalized attention scores (1.0 and 0.958) are highlighted in the figure (we perform min-max normalization on the scores). The substructure containing the two nodes is assigned with an attention score of 0.945.

As mentioned in Section 3.3.2, there is an attention layer in GCN to learn importance of each node (atom or substructure). By outputting the attention score for each node, we can observe the focus of the model for predicting DTI. Figure 9 shows an example. The atoms with the highest attention scores are highlighted. The two atoms, C (id = 13) and N (id = 14) obtain normalized attention scores of 1.0 and 0.958, respectively. Moreover, their belonging substructure also received a very high score, 0.945. Note that there exists quinazoline scaffold where these two atoms are located, in the molecule structure. According to [40], quinazoline ring system is considered to be the ‘master key’ in anticonvulsant therapy, because it constitutes the basic scaffold of many common anticonvulsant drugs. In fact, many structures bearing such quinazolinone scaffold exhibit potent anticonvulsant property, as shown in [40].

Furthermore, it is also mentioned by [41] that 4(3H)-Quinazolinone (with a carbonyl attached next to the marked atom N) with its derivatives possess a wide range of biological properties viz. anticancer, antibacterial, antitubercular, antifungal, anti-HIV, anticonvulsant, anti-inflammatory and analgesic activities. In this case, discovery of new antibacterial agents can be accelerated by effectively using quinazoline scaffold.

This result suggests that besides prediction, our model may reveal important bio-chemical properties of interactions between nodes or substructures, which could provide helpful insight and guidance in drug discovery.

## 7. Discussion

In this paper, we propose a new model called EmbedDTI for the prediction of drug-target interactions. The major goal of EmbedDTI is to enrich the representation of input target and compound information, to improve the prediction performance. The contributions of this study can be summarized in the following.

- To exploit abundant structural information from drugs, we model each drug molecule as both a graph of atoms and a graph of substructures (groups of nodes). And we propose algorithms for segmenting out the substructures and extracting their features. The experimental results show that the two-level graph representation contributes to the performance improved significantly.
- To fully use protein sequence information, we pre-train amino acid sequences via a large database using word embedding methods from natural language processing. The pre-trained embeddings are dense continuous vectors, which can represent the latent semantic correlation between amino acids. Moreover, a deep CNN is further employed to learn high-level abstract features of proteins. The enhanced protein representation also improves model performance.
- To interpret the learning ability of EmbedDTI, we add an attention mechanism to the GCN for learning atom-based graphs and substructure-based graphs. Different attention weights are assigned to the nodes in the molecule graph to evaluate their contributions. It can recognize important nodes as well as their interactions in the graphs, which provide useful hints in drug discovery.

As a result, two levels of molecule representation have better performance than single graph representation. Benefiting from the pretraining method, the word embedding method captures abundant amino acid information. In addition, we further discuss the interpretability of attention mechanism in drugs bearing the quinazolinone ring.

Although our proposed model has a better performance on the DTI prediction problem, there is still room for improvement. As a future work, we will design more effective algorithms incorporated with prior knowledge in the field of biochemistry to identify substructures with chemical properties. In addition, we will consider a better combination strategy of different levels of representation information instead of a simple concatenation.

## Figures and Tables

**Table 1:** Summary of Davis and KIBA datasets.

Dataset	# of Compounds	# of Proteins	# of DT Interactions
Davis	68	442	30,056
KIBA	2111	229	118,254

**Table 2:** Parameter setting for EmbedDTI *.

Parameters	Value
Batch size	512
Learning rate	0.0005
# epoch	1500
Dropout	0.2
Optimizer	Adam
# filters of the 3 layers in CNN	1000, 256, 32
Filter sizes of the 3 layers in CNN	8, 8, 3
Input Dim. of the 3 layers in GCN	*N*, *N*, 2N
Output Dim. of the 3 layers in GCN	*N*, 2N, 4N
# hidden units in final FC layers	1024, 512
Max length of protein sequences	1000

* *N* represents number of features.

**Table 3:** Comparison of MSE and CI scores on Davis test set *.

Models	Protein Rep.	Drug Pep.	MSE	CI
Baseline Models				
KronRLS	Smith-Waterman	Pubchem-Sim	0.379	0.871
SimBoost	Smith-Waterman	Pubchem-Sim	0.282	0.872
DeepDTA	1D	1D	0.261	0.878
WideDTA	1D + PDM	1D + LMCS	0.262	0.886
GraphDTA_GCN	1D	Graph	0.254	0.880
Our Proposed Models				
EmbedDTI_noPre	1D	Graph + Graph	0.236	0.892
EmbedDTI_noSub	1D	Graph	0.235	0.896
EmbedDTI_noAttn	1D	Graph + Graph	0.233	0.898
EmbedDTI	1D	Graph + Graph	**0.230**	**0.900**

Note: Rep. is short for representation. The best results are shown in bold.

**Table 4:** The MSE and CI scores of the KIBA test dataset comparision.

Models	Protein Rep.	Drug Rep.	MSE	CI
Baseline Models				
KronRLS	Smith-Waterman	Pubchem-Sim	0.411	0.782
SimBoost	Smith-Waterman	Pubchem-Sim	0.222	0.836
DeepDTA	1D	1D	0.194	0.863
WideDTA	1D + PDM	1D + LMCS	0.179	0.875
GraphDTA_GCN	1D	Graph	0.139	0.889
Our Proposed Models				
EmbedDTI_noPre	1D	Graph + Graph	0.134	0.896
EmbedDTI_noSub	1D	Graph	0.134	0.893
EmbedDTI_noAttn	1D	Graph + Graph	**0.131**	**0.901**
EmbedDTI	1D	Graph + Graph	0.133	0.897

Note: Rep. is short for representation. The best results are shown in bold.

**Table 5:** List of candidate SMILES sequences after virtual screening by EmbedDTI. Compounds are ranked by the prediction score $P_{i}$ (from low to high, the lower the better).

Rank Index	Canonical SMILES
1	Oc1ccc(-c2cncc(C(c3nc4c(C5NC6CCC5C6)cccc4[nH]3)c3cccc4ocnc34)c2)cc1
2	NC1CCCN(c2ccccc2S(=O)(=O)c2cc(C=Cc3ccccc3)cc(Cc3ccc4c(c3)OCO4)c2)C1
3	[O-]C1CNCCC1C1COc2ccc(CN3CCOCC3c3cnc4ccc(F)c(C(F)(F)F)c4c3)cc2O1
4	C=Cc1ccc(-c2cc(NC(=O)[O-])nc(-c3ccc(C4CC(=O)N(F)C4c4ccc(F)cc4)cc3)n2)cc1F
5	CC(=O)N1CCC(c2cccc(NNc3cc(Cl)cc(C4OCCC(C(=O)N5CCCCCC5)C4F)c3)c2)CC1
6	Oc1cnc(C2COC(c3ccc(Cl)c4c3OCC(c3cc(F)c(F)c5c3OCO5)O4)C(F)C2O)c(F)c1
7	O=C(C1CCc2cc(Nc3cc([O-])c(F)c(C4CN(c5ccc(F)cc5)CCO4)c3)cc(F)c21)N1CCNCC1
8	Fc1cc(Cc2ccc(-c3nc4ccc(F)c(F)c4s3)cc2)ccc1Nc1ccccc1-c1ccccc1
9	[O-]c1ccc(Nc2ccc(Cc3nc(-c4cccnc4)no3)c(Cc3cc(F)cc(-c4nnc([O-])o4)c3)c2)cc1
10	OC1C=C(c2cccc(C(F)(F)F)c2)CC(C2CCNC(C3CCOC(c4ccccn4)C3)C2)C1

**Table 6:** Metric list of candidate compounds after virtual screening by EmbedDTI. Compounds are indexed following the ranking order (prediction score $P_{i}$ from low to high, the lower the better).

Rank Index	Prediction Score	QED Score	SA Score	Docking Score (by SMINA)
1	−6.68	0.24	0.55	−12.44
2	−6.59	0.29	0.76	−12.68
3	−6.20	0.50	0.61	−12.38
4	−6.06	0.34	0.67	−12.68
5	−5.97	0.40	0.68	−12.42
6	−5.89	0.45	0.56	−12.62
7	−5.86	0.50	0.66	−12.43
8	−5.59	0.24	0.84	−12.32
9	−5.44	0.30	0.73	−12.31
10	−5.30	0.57	0.61	−12.34

## Data Availability

The source code are publicly available at https://github.com/Aurora-yuan/EmbedDTI accessed on 26 November 2021.

## Supplementary Materials

The following are available online at https://www.mdpi.com/article/10.3390/biom11121783/s1, Figure S1: Visualization of the first candidate molecule binding into specific pocket in chain A of K-Ras, Figure S2: Visualization of the second candidate molecule binding into specific pocket in chain A of K-Ras. Hydrogen bonds are highlighted by green lines, Figure S3: Visualization of the third candidate molecule binding into specific pocket in chain A of K-Ras. Hydrogen bonds are highlighted by green lines, Figure S4: Visualization of the fourth candidate molecule binding into specific pocket in chain A of K-Ras. Hydrogen bonds are highlighted by green lines, Figure S5: Visualization of the fifth candidate molecule binding into specific pocket in chain A of K-Ras. Hydrogen bonds are highlighted by green lines, Figure S6: Visualization of the sixth candidate molecule binding into specific pocket in chain A of K-Ras. Hydrogen bonds are highlighted by green lines, Figure S7: Visualization of the seventh candidate molecule binding into specific pocket in chain A of K-Ras. Hydrogen bonds are highlighted by green lines, Figure S8: Visualization of the eighth candidate molecule binding into specific pocket in chain A of K-Ras. Hydrogen bonds are highlighted by green lines, Figure S9: Visualization of the ninth candidate molecule binding into specific pocket in chain A of K-Ras. Hydrogen bonds are highlighted by green lines, Figure S10: Visualization of the tenth candidate molecule binding into specific pocket in chain A of K-Ras. Hydrogen bonds are highlighted by green lines.
