# Supplementary material for: EmbedDTI: Enhancing the Molecular Representations via Sequence Embedding and Graph Convolutional Network for the Prediction of Drug-Target Interaction
Source: Biomolecules. 2021 Nov 29;11(12):1783. doi: 10.3390/biom11121783 (PMC8698792; doi:10.3390/biom11121783)
Supplement: Supplementary file 1 [file biomolecules-11-01783-s001.zip › biomolecules-1444541-supplementary.pdf]

Supplementary Materials

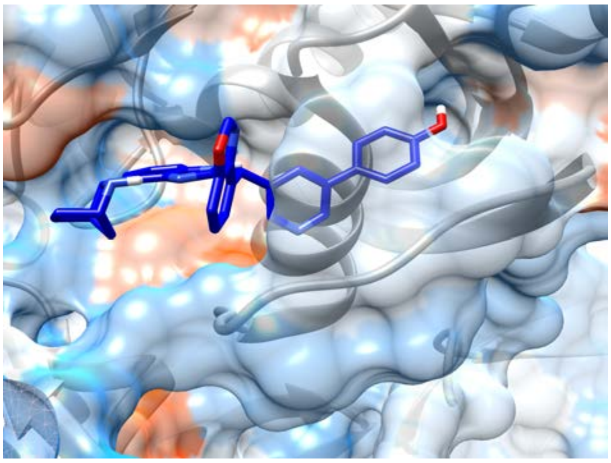

Figure S1. Visualization of the first candidate molecule binding into specific pocket in chain A of K-Ras.

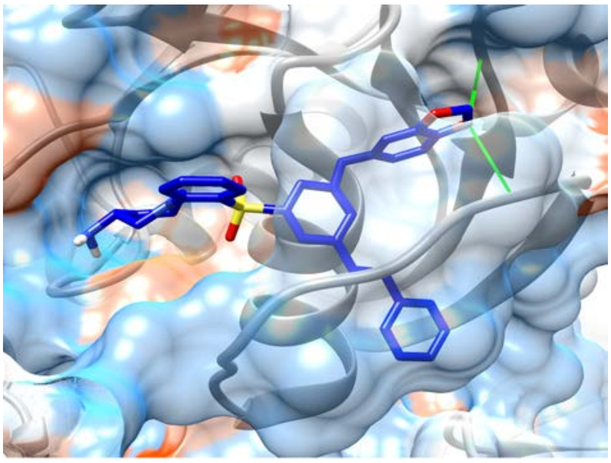

Figure S2. Visualization of the second candidate molecule binding into specific pocket in chain A of K-Ras. Hydrogen bonds are highlighted by green lines.

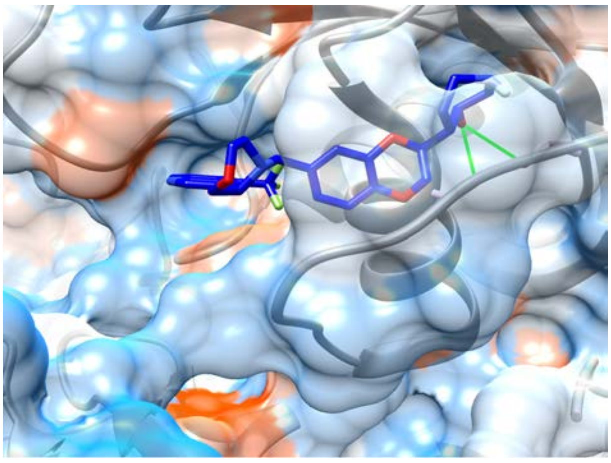

Figure S3. Visualization of the third candidate molecule binding into specific pocket in chain A of K-Ras. Hydrogen bonds are highlighted by green lines.

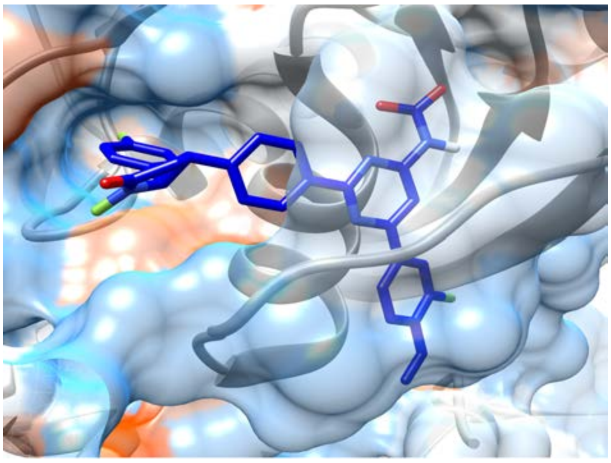

Figure S4. Visualization of the fourth candidate molecule binding into specific pocket in chain A of K-Ras. Hydrogen bonds are highlighted by green lines.

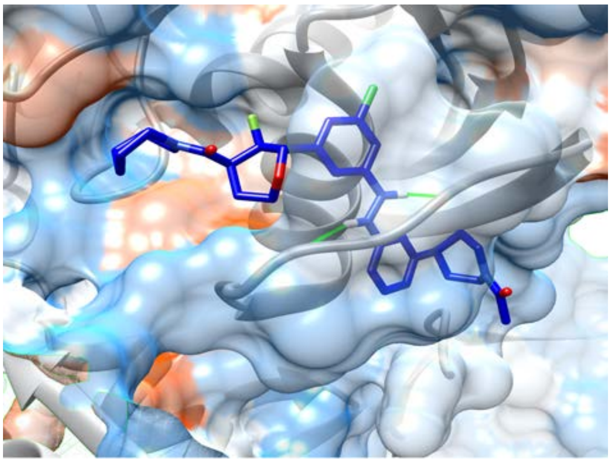

Figure S5. Visualization of the fifth candidate molecule binding into specific pocket in chain A of K-Ras. Hydrogen bonds are highlighted by green lines.

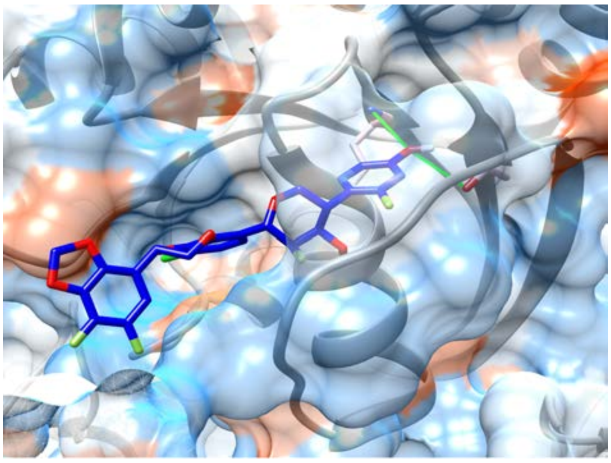

Figure S6. Visualization of the sixth candidate molecule binding into specific pocket in chain A of K-Ras. Hydrogen bonds are highlighted by green lines.

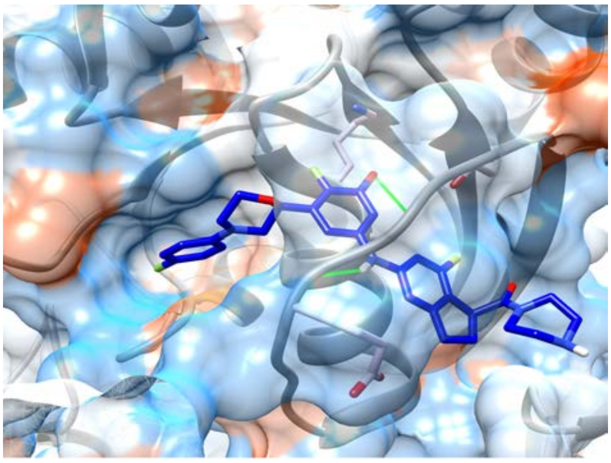

Figure S7. Visualization of the seventh candidate molecule binding into specific pocket in chain A of K-Ras. Hydrogen bonds are highlighted by green lines.

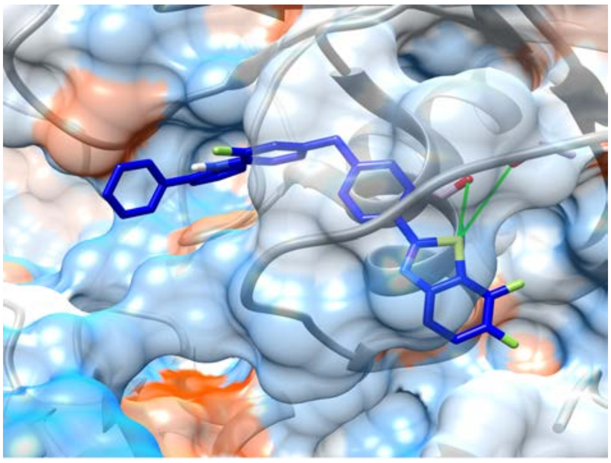

Figure S8. Visualization of the eighth candidate molecule binding into specific pocket in chain A of K-Ras. Hydrogen bonds are highlighted by green lines.

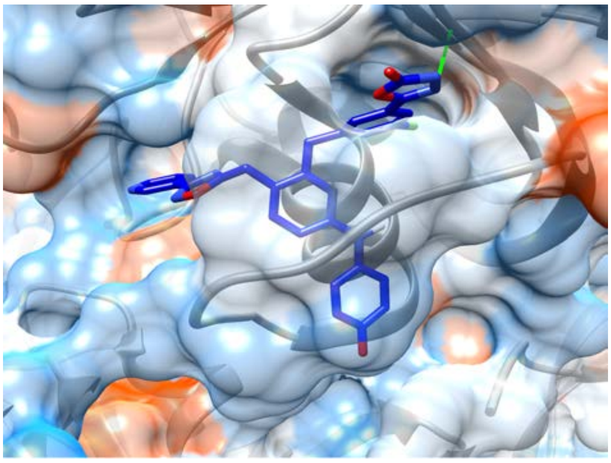

Figure S9. Visualization of the ninth candidate molecule binding into specific pocket in chain A of K-Ras. Hydrogen bonds are highlighted by green lines.

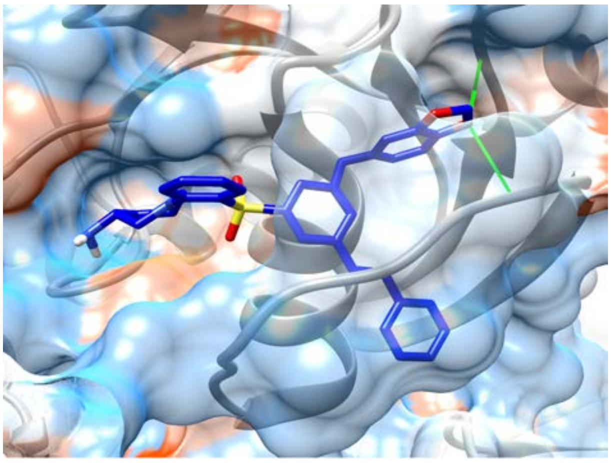

Figure S10. Visualization of the tenth candidate molecule binding into specific pocket in chain A of K-Ras. Hydrogen bonds are highlighted by green lines.
